# Rumination, anxiety, depressive symptoms and subsequent depression in adolescents at risk for psychopathology: a longitudinal cohort study

**DOI:** 10.1186/1471-244X-13-250

**Published:** 2013-10-08

**Authors:** Paul O Wilkinson, Tim J Croudace, Ian M Goodyer

**Affiliations:** 1 Department of Psychiatry, University of Cambridge, Cambridge, UK; 2 Cambridgeshire and Peterborough NHS Foundation Trust, Cambridge, UK; 3 University of York, York, UK

**Keywords:** Depression, Anxiety, Rumination, Factor analysis, Adolescence, Psychometrics

## Abstract

**Background:**

A ruminative style of responding to low mood is associated with subsequent high depressive symptoms and depressive disorder in children, adolescents and adults. Scores on self-report rumination scales correlate strongly with scores on anxiety and depression symptom scales. This may confound any associations between rumination and subsequent depression.

**Methods:**

Our sample comprised 658 healthy adolescents at elevated risk for psychopathology. This study applied ordinal item (non-linear) factor analysis to pooled items from three self-report questionnaires to explore whether there were separate, but correlated, constructs of rumination, depression and anxiety. It then tested whether rumination independently predicted depressive disorder and depressive symptoms over the subsequent 12 months, after adjusting for confounding variables.

**Results:**

We identified a single rumination factor, which was correlated with factors representing cognitive symptoms of depression, somatic symptoms of depression and anxiety symptoms; and one factor representing adaptive responses to low mood. Elevated rumination scores predicted onset of depressive disorders over the subsequent year (p = 0.035), and levels of depressive symptoms 12 months later (p < 0.0005), after adjustment for prior levels of depressive and anxiety symptoms.

**Conclusion:**

High rumination predicts onset of depressive disorder in healthy adolescents. Therapy that reduces rumination and increases distraction/problem-solving may reduce onset and relapse rates of depression.

## Background

A mood-related ruminative response style refers to how a person, when dysphoric, focuses attention on his or her symptoms, and their ‘potential causes, implications and consequences’ [1]. Rumination is frequently studied alongside affective psychopathology and is usually assessed using the multi-item Response Styles Questionnaire (RSQ) [2]. High rumination scores on the RSQ predicts higher future depressive symptoms [3] and DSM-defined major depressive episodes in child and adolescent samples [4-6].

This rumination-depression association appears to be stronger in studies of adolescents than of children [3]. This may be due to greater exposure to negative stressors from the age of 13; this is relevant because rumination may moderate the depressogenic effect of stressors [6]. Alternatively this may reflect differences in other cognitive vulnerability factors that manifest differentially with increasing age [7]. Another possible explanation is that it is the effects of puberty (with the change in hormonal milieu) that increases the depressogenic effect of rumination, rather than age itself. This has not been tested to date.

### Potential confounders to rumination-depression associations

Self-rated rumination is strongly correlated with concurrent depressive [3] and anxiety [8-10] symptoms, which are themselves strong predictors of future depressive symptoms and disorder [1]. This may confound the associations found in above studies between rumination and future depression. It has been argued that this is partly because high levels of depressive symptoms would themselves make scores on some rumination items higher. For example a score on the RSQ item ‘I think about how sad I feel’ may be high either because of a high tendency to ruminate on low mood, or the fact that the person is currently very sad so thinks about this a lot [11]. Therefore high RSQ scores may be associated with future onset of depression because high concurrent depressive symptoms lead to both high RSQ scores and high risk of depression; and it may be the case that the cognitive style of ruminating has no effect on depressive symptoms.

Two methods have been used to control for such potential depressive symptom –rumination confounding. Firstly, some studies have statistically controlled for baseline depressive symptoms. For example, controlling for depressive symptoms attenuated the correlation between baseline rumination and follow-up depressive symptoms from r = 0.3 to r = 0.07 (95% CI 0.03-0.11) in a meta-analysis of childhood/adolescence studies [3]. Two studies in adolescents have found that rumination scores are associated with future onset of depressive disorder, even when controlling for concurrent depressive symptoms [4,6]. No studies have controlled for prior levels of anxiety in addition.

The second method has been to restrict use of items to those from the rumination questionnaire that are likely to measure actual rumination, as opposed to items that are strongly influenced by current depressive symptoms. Often such studies have used linear factor analysis methods or principal components analysis (PCA) to explore multiple dimensions among item sets. Initial studies in community-recruited adults [9,11,12] identified a ‘brooding’ factor/principal component, which was more strongly associated with current and/or future depressive symptoms than any other dimension of the RSQ (in particular a ‘reflecting’ factor/PC). However, while some studies in adolescents found a similar two factor structure of the RSQ [10,13], one found only a single factor [14].

In this study a third methodological approach is considered that might better separate the rumination construct from depressive and anxiety symptoms. If some items from the rumination questionnaire are in fact measures of depressive symptoms, they would be expected to correlate strongly with items from questionnaires measuring depressive symptoms. If items from both the rumination and depressive questionnaires were entered into the same factor analysis, we could identify whether such items (ie ‘depression’ items from the rumination questionnaire) load better with the depressive symptom items, the rumination items, or are in fact part of a separate construct. Likewise, the addition of items from an anxiety questionnaire would identify rumination questionnaire items that load better with anxiety symptoms. It is also possible that items from all questionnaires would inter-correlate strongly with each other, and the factor analysis would suggest just a single common factor as a parsimonious solution. In this case, items would be best seen as measuring one common construct; this construct could be termed ‘negative cognitions’, and would be a risk factor for future depression (and possibly anxiety). Studies to date have made the prior assumption that items from rumination, depressive symptom and anxiety symptom questionnaires measure separate constructs, so should more appropiately be analysed separately. We propose that entering all items into a pooled factor analysis could explore whether this assumption is likely to be correct.

### Distraction and problem-solving

In addition to the features already discussed, the RSQ also contains two further sub-scales called distraction and problem-solving, which are thought to be adaptive responses to low mood. Factor analysis suggests that items from both scales load onto a single factor [15,16].

High levels of distraction and problem-solving have been found to be associated with reduced future depressive symptoms in community [15] and high-risk [16] samples of children and adolescents, controlling for prior depressive symptoms. In addition, the ratio of rumination to distraction/problem-solving is associated with increased depressive symptoms at follow-up, suggesting that high levels of distraction/problem-solving mitigate some of the effects of rumination [15,16]. As high distraction/problem-solving in itself probably leads to reduced depression risk (rather than just reducing the effects of high rumination), a linear ratio approach has been considered a better way to model this data than a rumination x distraction interaction [16].

### Goals of the current study

Our analysis consisted of two phases. In the first phase, we investigated the factor structure of a joint set of items from all three self report questionnaires purporting to measure depressive symptoms, anxiety symptoms and rumination. We hypothesized that this would either identify all items measure one underlying ‘negative cognitions’ construct; or alternatively identify multiple separate constructs, including rumination (and possibly different forms of rumination). This factor analysis would ideally assign each item from the pooled item set to the appropriate construct.

In our second phase, we hypothesized that high rumination would predict onset of a depressive episode over the subsequent 12 months and high depressive symptoms 12 months after baseline; this would be true controlling for confounding from baseline depressive and anxiety symptoms. As the first phase would identify whether RDQ (rumination questionnaire) scale items loaded best with the rest of the RDQ questionnaire or other questionnaires, the a priori plan was to only include scores from items found to load with ‘rumination’ to make up this total rumination scale for this analysis. We also hypothesized that a high ratio of rumination to distracting/problem-solving response styles would be associated with a higher risk of depression onset/symptoms, indicating that these adaptive response styles partially mitigate the effects of rumination. We hypothesized that effects of rumination were stronger for mid/post-pubertal adolescents than pre/early-pubertal adolescents, and tested this with pubertal stage x rumination interaction terms. To disentangle effects of age and puberty, we also tested age x rumination interactions.

## Methods

### Participants

A sample of 658 healthy adolescents aged 12 to 16 years was recruited from Cambridgeshire secondary schools from 1999 to 2002. All were currently mentally and physically well, and had no past episodes of depression.

We recruited a risk-enriched sample to increase the predicted onset of psychiatric disorders (in particular depression), to increase the power of the study. Adolescents and their parents completed short, screening checklists at entry, which asked about family psychiatric history and social adversity. Mothers completed the EAS Temperament Survey [17]. Participants were included if they had a parent with a lifetime psychiatric history; or 2 or more of the following:

–2 lifetime bereavements

–EAS emotionality > 17

–chronic (> 6 months) marital dysharmony or parental separation

–2 recent undesirable life events

–difficulties with family or friendships focused on the adolescent.

We have established that this risk profile is associated with a three to fivefold increase in the risk for onset of an episode of major depression over one year [18]. We also recruited a smaller low risk sample in the present study. In addition, we applied the same risk criteria to an independent community sample of 1089 adolescents recruited without any enrichment by risk factors. In both samples, depressive symptoms were significantly higher in high-risk than low-risk adolescents; and depressive symptoms were similar in the high-risk groups of each study [19].

### Measures

#### Mood-related response style

The Responses to Depression Questionnaire, RDQ, is a modified version of the Response Styles Questionnaire [1], with wording of a small number of items slightly altered to make it more appropriate and simpler to understand for adolescents [20]. Additional file 1 shows all items in full. It comprises 39 items asking participants what they habitually think, do or feel when they experience low mood. Each item response is scored using four response levels (0 = almost never, 1 = sometimes, 2 = often, and 3 = almost always). There are four groups of items that are scored as sub-scales, which estimate the tendency to use different mental strategies for dealing with low mood: rumination, distraction, problem-solving and dangerous (acts). The dangerous acts scale has shown poor psychometrics and validity, therefore items from this scale were not entered into the factor analysis, nor analysed in this study. The RDQ contains 5 out of 6 items labeled as ‘brooding’ and all four ‘reflecting’ items in the Burwell and Shirk (2007) principal component analysis study in adolescents.

#### Depressive symptoms

The self-rated Mood and Feelings Questionnaire, MFQ, was completed at study entry and at 12-month follow-up. The MFQ comprises 33-items measuring depressive symptoms [21]. Re-test reliability and criterion validity are often reported to be high [22] and marginal reliability estimates from our study exceeded .90. A four-point ordinal response scale was adopted instead of the original three categories, because of its embedding along with other assessments in a common format, the “Young Person Questionnaire (YPQ)”. Items were scored from 0–3 (never, sometimes, mostly, always). Responses in the “mostly” and “always” categories were combined, to yield three response levels whose prevalence combined was found to be highly similar to the high upper rating category of the original version.

#### Anxiety symptoms

The self-rated Revised Children’s Manifest Anxiety Scale, RCMAS, contains 28 anxiety items. It also has high reported internal consistency and reliability [23] with marginal reliability estimates from our study exceeding .85. The RCMAS anxiety items were included as part of the YPQ and each item was scored from 0–3 (never, sometimes, mostly, always). Since 5 items from the MFQ and RCMAS had very similar wording, only the MFQ item for these questions was included; these were completed within the MFQ item block in the YPQ. Participants’ scores were included if they had at least 50% of items completed within each of the RDQ, MFQ and RCMAS.

#### Diagnoses

The Lifetime Schedule for Affective Disorders and Schizophrenia for Adolescents, K-SADS-L, is a semi-structured interview which can be used to ascertain lifetime and current DSM-IV diagnoses of psychiatric disorders [24]. It was used to exclude participants with psychiatric disorder at baseline, and establish onsets of depressive disorders during 12 month follow-up. Depressive disorders were defined as DSM-IV major depressive episode (MDE) or minor depressive episode (3/4 symptoms together with significant psychosocial impairment, defined as a Children’s’ Global Assessment Score of <60). Raters were graduate research assistants who had extensive training. All possible diagnoses of disorder were discussed at consensus meetings with one of the senior investigators, to ensure reliability.

#### Pubertal stage assessment

Pubertal stage was assessed using schematic drawings of secondary sex characteristics associated with the five Tanner stages of pubertal development [25]. Sketches were adapted from Greydanus and Shearin [26]. Participants were provided with gender appropriate sketches and were asked to select which of the sketches looked most like them. In view of the sample size and to allow adequate power, participants were a priori dichotomized into being either pre/early-puberty (Tanner stages 1, 2) or mid/post-puberty (Tanner stages 3–5).

### Procedure

Adolescents were interviewed at study entry and 12 month follow-up. In a constant order, MFQ, K-SADS-PL, RCMAS then RDQ were administered at study entry. K-SADS-PL and MFQ were administered at 12 month follow-up.

### Statistical analysis

#### Factor analysis of cognitive styles

We initially carried out exploratory factor analysis (EFA) for ordinal items using MPlus, Version 5.2 [27] via a latent probit item response model approach, of all items from the RDQ, MFQ and RCMAS. MPlus allows for accurate results to be given if some data is missing. As items are scored on an ordinal scale, these were treated as categorical, and a polychoric correlation matrix was used; parameters reported are the weighted least square parameter estimates using a diagonal weight matrix (WLSMV). Linear factor models for ordinal psychopathology and self-reported mood and cognition data can yield misleading factor solutions, such as factors defined by prevalence and skew rather than content and other distortions of the true factor structure due to aspects of scale use. EFA solutions were rotated using Promax (correlated factors) to aid interpretation. Confirmatory factor analysis was performed using MPlus Version 5.2 to further examine the fit of the factor structures estimated by EFA in the same sample. Items loading highest on each factor were summed to give scale scores for each factor and these were used in subsequent analyses, to aid application of these findings to clinicians and researchers using these questionnaires. Where fewer than 50% of items were missing for each scale for a participant, missing values were imputed as being the average for that scale from present data.

#### Predicting onset of disorders and symptoms

We tested whether scale scores were associated with risk of onset of a new depressive episode over the following 12 months, using Stata 11 [28]. For our prediction analyses, we related each item and scale score to the (binary) diagnostic outcomes. To provide a non-parametric estimate of the overall strength of this predictive relationship, we used Receiver Operating Characteristic (ROC) curve methods (roccomp on Stata 11). An area under the curve (AUC) AUC of 0.5 suggests the predictive properties of an item are no better than chance. AUC > 0.5 suggests that presence of an item predicts the outcome of interest. AUC < 0.5 suggests that presence of an item reduces the risk of the outcome of interest. The roccomp function was used to compare strength of association between predictors and depression onset.

We tested whether our scales predicted depressive symptoms (measured by the MFQ) at 12 month follow-up, using correlation statistics. We decided a priori to use the total MFQ score as it is accepted as a valid single measure of total depressive symptoms, and so results would be more understandable. We used Fisher r-to-z transformation to test whether correlation coefficients were significantly different [29].

Simple ROC methods do not allow for adjustment by other variables. We used multiple logistic and linear regression to test whether rumination at baseline predicts onset of depression and 12 month depressive symptoms, controlling for the possible confounding variables of baseline depressive and anxiety symptoms and gender, age and pubertal stage; interaction terms were used to test whether effects of rumination were different between ages and between pubertal groups.

Written informed consent for clinical and questionnaire assessment and follow-up was given by each participant together with one of their parents. Ethical approval was given by the Cambridge Local Research Ethics Committee.

## Results

Descriptive statistics for the demographics of the sample and questionnaire scores are presented in Table 1. 645/658 (98%) participants had all 92 questionnaire items completed, 656/658 (99.7%) had 91 or 92 items completed. The remaining two participants had greater than 80% of total items completed and at least 50% of items of each questionnaire completed.

**Table 1:** Characteristics of final sample at study entry and MFQ at follow-up

	**Boys (n = 338, 57%)**	**Girls (n = 260, 43%)**
Age	13.7 (1.2)	13.7 (1.1)
Pubertal status at entry		
Pre/early-puberty	66 (19.6%)	19 (7.4%)
Mid/post-puberty	271 (80.4%)	238 (92.6%)
Initial scores at study entry		
MFQ	17.2 (8.7)	19.1 (9.4)
Rumination	13.8 (9.3)	17.3 (10.4)
Distraction	12.1 (5.2)	13.4 (6.3)
Problem-solving	3.4 (2.3)	4.5 (2.7)
RCMAS	16.6 (8.0)	17.7 (8.7)
Follow-up at 12 months		
MFQ	14.0 (8.4)	16.5 (10.1)

All entries are mean (standard deviation) unless stated otherwise.

MFQ = Mood and Feelings Questionnaire, RCMAS = Revised Children’s Manifest Anxiety Scale.

### Factor analysis of the RDQ, MFQ and RCMAS

#### Exploratory Factor Analysis (EFA) of RDQ, MFQ and RCMAS items

The six largest eigenvalues from the EFA were 20.6, 6.9, 4.7, 3.8, 2.5, 2.1 and 2.0. Inspection of the scree plot suggested a four or five factor solution. The six factor solution contained one extra factor with relatively low determinacy (0.885); no items loaded highest on this extra factor, and all other items loaded onto the same factors as in the five factor solution.

#### Confirmatory factor analysis (CFA) of RDQ, MFQ and RCMAS items

The four and five factor solutions were entered estimated as separate CFAs. Model fit was slightly better for the five than four factor solution (four factor: CFI 0.840, TLI 0.901, RMSEA 0.064; five factor: CFI 0.850, TLI 0.908; RMSEA 0.062). The five factor model was chosen both because its latent structure appeared more interpretable and because under-factoring is more likely to lead to interpretation problems than over-factoring [30]. Additional file 2 shows results of the five factor CFA. Item scores were summed for each of the five factors for inclusion in regression analyses.

There was a strong and interpretable pattern in the Promax-rotated results of the five factor solution from the joint factor analysis of pooled items from the RDQ (Responses to Depression Questionnaire), MFQ (Mood and Feelings Questionnaire) and RCMAS (Revised Children’s Manifest Anxiety Scale). 19 out of 21 rumination items loaded highest on factor 4 (‘rumination’ factor); 21 MFQ items, referring to emotional or cognitive symptoms of depression, loaded onto factor one (‘cognitive’ factor); 11 MFQ items, referring to more physiological and ‘melancholic’ symptoms of depression including poor concentration and anhedonia, 2 RDQ rumination items (‘I think about how hard it is to concentrate’ and ‘I think about my feelings of tiredness’) and one RCMAS-only item (‘It was hard for me to keep my mind on my schoolwork’) loaded highest on factor two (‘somatic’ factor); 22 out of 23 RCMAS-only items loaded highest on factor 3 (‘anxiety’ factor). All distraction and problem-solving items from the RDQ loaded highest on factor 5 (‘adaptive’ factor). Of the 5 RCMAS items also found in (and completed within) the MFQ, 3 (referring to primarily cognitive/emotional symptoms) loaded highest on cognitive factor and 2 (referring to primarily physiological symptoms) loaded highest on somatic factor. All future analyses use the sum of the scores from items loading best on each of the five factors (ie for rumination, we use the sum of the 19 items that load best onto the rumination factor).

Inter-factor correlations for factor sum scores are presented in Table 2, demonstrating moderate-high associations between all factors except adaptive.

**Table 2:** Pairwise correlations between factor item totals at study entry and MFQ scores at 1 year follow-up

	**1**	**2**	**3**	**4**	**5**	**6**	**7**
1. Rumination	1						
2. Adaptive	0.24*	1					
3. Cognitive	0.42*	0.03	1				
4. Somatic	0.41*	0.10*	0.54*	1			
5. Anxiety	0.54*	0.05	0.55*	0.55*	1		
6. Rumination : Adaptive Ratio	0.55*	−0.39*	0.25*	0.20*	0.35*	1	
7. MFQ at 12 months	0.49*	0.10*	0.55*	0.51*	0.60*	0.26*	1

* p < 0.05.

Items 1–6 are total factor scores, as described in the text.

### Association between baseline rumination and onset of DSM-IV depressive episode over the subsequent 12 months

#### Prediction of depression onset

12 month follow-up data was available for 598 out of 658 (91%) participants. Younger cohort members were less likely to be retained in the study [mean(sd) 13.7(1.1) vs 14.6(1.2), *Z* = 5.5, p < 0.0005]. There were no major differences in attrition by sex, pubertal group nor initial questionnaire scores (all p > 0.15). 62 (10.4%) had onset of a depressive episode.

Additional file 1 shows the ROC area under the curve (AUC) estimates for predicting binary outcomes capturing depression onset over 12 months from all RDQ, MFQ and RCMAS items. Table 3 compares the AUCs for the sum scores from our factors. Rumination, cognitive, somatic and anxiety factors were all significantly associated with risk of depression onset. High rumination:adaptive ratio was significantly associated with risk of depression onset, although this association was not significantly different to rumination alone (p = 0.8). There was no significant difference in the predictive effects on risk of depression between cognitive and somatic sub-scale factors (p = 0.2).

**Table 3:** Prediction of depression outcomes over one year from factor scores of mid/post-pubertal participants

	**Area under the curve**	**Asymptotic 95% confidence interval**
*Factors Derived from Five Factor EFA*		
Rumination	0.681	0.612–0.750 *
Adaptive	0.472	0.393–0.551
Cognitive	0.710	0.649–0.772 *
Somatic	0.671	0.599–0.742 *
Anxiety	0.681	0.609–0.753 *
Rumination : Adaptive Ratio	0.687	0.617–0.756 *

* 95% confidence interval of AUC does not include 0.5

Only three pre/early pubertal participants had depression onset, so we were unable to test whether puberty moderated other risk factors. Age x rumination interaction was non-significant (p = 0.3).

Results of our multiple logistic regression for significant independent predictors of depression onset are shown in Table 4. Higher rumination was independently associated with risk of clinical depression episode onset (OR *=* 1.04, p = 0.035). There was a trend for higher adaptive factor scores (distraction/problem-solving) to be associated with lower risk of depression onset (OR = 0.96, p = 0.053). Cognitive, somatic, anxiety, gender, age and pubertal group were not significantly independently associated with risk of depression onset.

**Table 4:** Multiple logistic regression analysis demonstrating the contribution of cognitive styles and initial symptom levels to liability of clinical depression episode onsets over 12 months

	**Coefficient**	**Odds ratio**	**95% CI of OR**	**p**
Rumination	0.03	1.04	1.00–1.07	0.035
Adaptive	−0.04	0.96	0.92–1.00	0.053
Cognitive	0.04	1.04	0.99–1.09	0.15
Somatic	0.07	1.08	0.99–1.17	0.071
Anxiety	0.02	1.02	0.97–1.07	0.5
Gender	0.33	1.40	0.77–2.54	0.3
Age (years)	−0.07	0.93	0.72–1.20	0.6
Pubertal group	1.12	3.08	0.88–10.7	0.077

The regression was repeated with the rumination:adaptive ratio included rather than separate rumination and adaptive factors. This rumination: adaptive ratio was significantly associated with risk of depression onset (OR = 1.25, p = 0.018). Model fit was marginally better for this regression (Akaike Information Criterion, AIC = 366.8) than the one with separate rumination and adaptive items (AIC = 367.6).

#### Prediction of depressive symptoms (MFQ Scores) at 12 month follow-up

Total MFQ scores at 12 months were available for 590 out of 658 (90%) participants. Table 2 demonstrates that our measures of baseline ruminative style, depressive symptoms and anxiety were strongly correlated with depressive symptoms at 12 months. High adaptive was correlated with lower depressive symptoms at 12 months. Rumination was more strongly correlated with depressive symptoms at 12 months than rumination:adaptive ratio (z = 7.4, p < 0.0001). There was no significant difference in the predictive effects on depressive symptoms between cognitive and somatic sub-scale factors (p = 0.4).

Table 5 shows the results of multiple linear regression with depressive symptoms at 12 months as outcome variable (total n = 584). Rumination (β = 0.14, p < 0.0005), cognitive, somatic and anxiety scores and female gender were significantly and independently associated with higher depressive symptoms at 12 months. The adaptive factor, pubertal group and age were not significantly associated with higher depressive symptoms at follow-up.

**Table 5:** Multiple linear regression analysis demonstrating the contribution of cognitive styles and baseline symptoms to MFQ score at 12 month follow-up

	**β Coefficient**	**95% CI of β**	**t**	**p**
Rumination	0.14	0.07–0.22	3.7	<0.0005
Adaptive	0.02	−0.05–0.10	0.6	0.5
Cognitive	0.34	0.22–0.46	5.6	<0.0005
Somatic	0.33	0.16–0.49	3.9	<0.0005
Anxiety	0.41	0.30–0.52	7.2	<0.0005
Gender	1.26	0.04–2.48	2.0	0.043
Age	0.11	−0.42–0.64	0.4	0.7
Pubertal status	−0.69	−2.45–1.06	−0.8	0.4

In separate regressions, there were no significant interaction effects for rumination with pubertal group nor age on depressive symptoms at 12 months (p > 0.5). Regressions were performed separately for pre/early-pubertal participants and mid/post-pubertal participants. Regression coefficients were similar for rumination in each (pre-early puberty: 0.13, mid-post puberty: 0.14).

## Discussion

### Factor structure of the Responses to Depression Questionnaire (RDQ), Mood and Feelings Questionnaire (MFQ) and Revised Children’s Manifest Anxiety Scale (RCMAS)

We found that most of the RDQ rumination and MFQ depression items loaded onto separate factors in our ordinal item exploratory factor analysis. This suggests that these questionnaires measure different, albeit significantly correlated constructs. We found that the MFQ items at baseline captured two important dimensions, one factor enumerating cognitive-emotional symptoms, the other more somatic-physical symptoms. One factor (anxiety) contained 22 RCMAS items. This appears to be a measure of anxiety symptoms, that is separate to (but correlated strongly with) rumination and depressive symptoms.

Unlike most previous research, including in adolescents [13], we found the rumination items in the RDQ scale to be essentially unidimensional. Only two RDQ items loaded better with MFQ items than the other RDQ items. In addition, supplementary analysis (available from the 1^st^ author on request) demonstrated no difference in predictive effect between ‘brooding’ and ‘reflecting’ items; it also demonstrated that total rumination was more strongly associated with future depressive disorder and depressive symptoms than total score of ‘brooding’ items. One other study in adolescents had a similar finding of unidimensionality [14]. One study demonstrated that while brooding and reflecting factors are both identified in healthy adults, they are not in depressed adults [31]. Another adult study demonstrated no difference in correlation between depressive symptoms and brooding or reflection [32]. Such contradictory evidence makes it unclear whether the RSQ should be treated as a unifactorial or multifactorial scale.

### Prediction of depressive disorder and depressive symptoms

This study has confirmed previous findings that rumination increases the risk of future depression onset, independently of baseline depressive symptoms. In addition, this study has demonstrated that this effect is independent of another potential confounder–baseline anxiety symptoms. This suggests that, as predicted, a ruminative style of responding to low mood does in itself make people more prone to developing depression, rather than simply being a proxy measure of depressive and/or anxiety symptoms. However, effects on future depression were only small-moderate, with a one point increase in the RDQ independently associated with an odds ratio of 1.04 for increased risk of onset of depression in the next year, and a total MFQ score 0.14 points higher.

Both the cognitive-emotional and somatic-physical subscales were associated with future depressive symptoms. While both sub-scales were associated with risk of onset of depressive disorder in univariate analysis, this became non-significant when controlling for other variables including rumination. The low number of depression onsets makes it possible that this is a type 2 error and so it is difficult to draw conclusions on the effects of these symptoms on risk of depressive disorder. Our findings do suggest that both sub-scales do have similar effects on future depressive symptoms/disorder, and therefore both types of depressive symptoms are important in indexing future risk.

### Distraction and problem-solving response styles

Our exploratory factor analysis results are highly similar to prior findings that distraction and problem-solving are most appropriately treated as a single factor. We found a trend for greater use of these adaptive strategies to confer resilience against risk of onset of depression. Rumination:adaptive ratio was not a stronger predictor of depression/depressive symptoms than Rumination alone, therefore we did not find evidence that Adaptive strategies significantly mitigated the effects of Rumination. However, we still consider that it is worthwhile to include these items in larger longitudinal research to explore whether they reduce depression risk over time.

### Pubertal stage and rumination

There was no evidence that either pubertal stage or age moderated the effects of rumination on depressive symptoms. This may reflect the relatively narrow age range of the sample (12–16) and the fact that more than 80% of the sample were in mid-late puberty. This suggests that a sample with a wider age range is needed to disentangle the effects of puberty and age on rumination.

### Limitations

A major limitation of this study is that the sample was of adolescents at risk for psychiatric disorder. It is possible that rumination only acts to moderate the effects of these particular risk factors on depression risk, and does not in itself cause depression. Therefore results may not be generalizable to the whole population. However, understanding the roles of these cognitive styles in those at high risk for depression will help us in developing appropriate interventions for adolescents at greatest risk.

Our study was also limited by the lack of power resultant from an incidence of depressive episode onset of only 10%, despite a risk-enriched sample. Our risk enrichment strategy was designed to increase numbers of cases of psychopathology in general, not just depression. This was partly because risk factors for depression also have significant effects on other disorders (particularly anxiety disorders). It is possible that a more depression-specific set of criteria (for example family history of depressive disorder and loss life events only) would have increased the number of onsets of depression. However, this may have reduced generalisability further.

Our sample was aged 12–16, with a significant majority of the sample being in mid-post puberty. This may explain why we were unable to find significant moderating effects of age or puberty on rumination.

The fit indices of our final model were some way below current recommendations, therefore some caution is needed when interpreting the factor structure (despite our tendency to interpret overfactored solutions). However, the five factor solution interpreted was better fitting than others considered. This also does not alter the prospective results that the sum score measure of rumination significantly predicted depressive disorder and depressive symptoms.

## Conclusion

This study has demonstrated that a ruminative style of responding to low mood is indeed unhelpful and increases the risk of developing depressive disorders, or promotes high symptom loads. Therapy that reduces rumination may thereby reduce the risk of subsequent emotional disorders. This study also provides some preliminary support for further investigating whether increasing the use of distraction/problem-solving response styles reduces risk of onset of depressive disorders. Cognitive-behavioural therapy (CBT) has been shown to reduce rumination in currently-depressed adolescents [33]. Rumination-focused CBT reduces both depressive symptoms and rumination in adults in partial remission from depression [34]. The current findings support further investigations into the efficacy and effectiveness of a psychological treatment that reduces ruminative thinking style and promotes cognitive resilience in adolescents at risk for or suffering from depressive disorders.

## Abbreviations

RDQ: Responses to depression questionnaire; MFQ: Mood and feelings questionnaire; RCMAS: Revised children’s manifest anxiety scale; K-SADS-L: Lifetime schedule for affective disorders and schizophrenia for adolescents; EFA: Exploratory factor analysis; ROC: Receiver operating characteristic; AUC: Area under the curve; OR: Odds ratio; AIC: Akaike information criterion; PEP: Pre/early-pubertal; MPP: Mid/post-pubertal.

## Competing interests

None of the authors have competing interests.

## Authors’ contributions

POW analysed the data and wrote the first draft of the manuscript. IMG designed the study and contributed to the writing of the manuscript. TJC supervised statistical analysis and contributed to the writing of the manuscript. All authors read and approved the final manuscript.

## Pre-publication history

The pre-publication history for this paper can be accessed here:

http://www.biomedcentral.com/1471-244X/13/250/prepub

## Supplementary Material

Additional file 1: Table S1 Exploratory Factor Analysis of Pooled RDQ, MFQ and RCMAS items. Click here for file

Additional file 2: Table S2 Five factor confirmatory factor analysis of pooled RDQ, MFQ and RCMAS items. Click here for file
