# Supplementary material for: Rumination, anxiety, depressive symptoms and subsequent depression in adolescents at risk for psychopathology: a longitudinal cohort study
Source: BMC Psychiatry. 2013 Oct 8;13:250. doi: 10.1186/1471-244X-13-250 (PMC3851454; doi:10.1186/1471-244X-13-250)
Supplement: Additional file 1: Table S1 — Exploratory Factor Analysis of Pooled RDQ, MFQ and RCMAS items. [file 1471-244X-13-250-S1.docx]

**Supplementary Table s1 Exploratory Factor Analysis of Pooled RDQ, MFQ and RCMAS items**

|  |  | **Factor**  **1**  *Cognitive* | **Factor 2**  *Somatic* | **Factor 3**  *Anxiety* | **Factor**  **4**  *Rumin-ation* | **Factor**  **5**  *Adaptive* | **Area**  **Under Curve** |
| --- | --- | --- | --- | --- | --- | --- | --- |
| **RDQ Items** | **RDQ**  **Sub-scale** |  |  |  |  |  | **AUC** |
| 1. I ask someone to help me overcome a problem | PS | 0.072 | -0.358 | 0.064 | 0.190 | **-0.467** | 0.526 |
| 2. I think about how alone I feel | Rum | 0.079 | 0.067 | 0.057 | **0.596** | 0.140 | 0.633* |
| 3. I think “I won’t be able to do my work because I feel so bad” | Rum | -0.017 | 0.298 | 0.177 | **0.313** | -0.033 | 0.552 |
| 4. I think about my feelings of tiredness | Rum | -0.189 | **0.306** | 0.193 | 0.283 | -0.069 | 0.624* |
| 5. I think about how hard it is to concentrate | Rum | -0.243 | **0.513** | 0.192 | 0.292 | -0.087 | 0.663* |
| 6. I try to find something positive or something I’ve learned in the situation | Dist | -0.156 | 0.159 | -0.083 | 0.111 | **-0.546** | 0.517 |
| 8. I think “I’m going to do something to make myself feel better” | Dist | 0.061 | 0.052 | -0.141 | 0.029 | **-0.637** | 0.478 |
| 9. I help someone else with something in order to distract myself | Dist | -0.003 | 0.018 | -0.027 | 0.177 | **-0.516** | 0.490 |
| 10. I think about how I don’t feel like doing anything | Rum | -0.119 | 0.304 | 0.111 | **0.522** | 0.092 | 0.589* |
| 11. I remind myself that these feelings won’t last | Dist | -0.073 | 0.049 | -0.159 | 0.209 | **-0.500** | 0.485 |
| 12. I think about recent events to try to understand why I am depressed | Rum | -0.093 | 0.118 | -0.065 | **0.618** | -0.159 | 0.617* |
| 13. I think about how I don’t seem to feel anything any more | Rum | -0.041 | 0.335 | 0.062 | **0.438** | 0.028 | 0.587* |
| 14. I think “Why can’t I get going?” | Rum | -0.182 | 0.317 | 0.150 | **0.377** | -0.156 | 0.591* |
| 15. I think “Why do I always react this way?” | Rum | -0.034 | 0.164 | 0.248 | **0.312** | -0.137 | 0.591* |
| 16. I go to a favourite place to get my mind off my feelings | Dist | 0.100 | 0.044 | -0.045 | 0.185 | **-0.370** | 0.500 |
| 17. I go away by myself and think about why I feel this way | Rum | 0.173 | -0.064 | -0.114 | **0.708** | -0.065 | 0.627* |
| 18. I talk it out with someone whose opinions I respect | PS | 0.058 | -0.332 | 0.049 | 0.184 | **-0.554** | 0.572* |
| 19. I think “I’ll concentrate on something other than how I feel” | Dist | -0.111 | -0.014 | -0.048 | 0.207 | **-0.524** | 0.500 |
| 20. I write down what I’m thinking and try to sort it out | Rum | 0.171 | -0.056 | 0.001 | **0.327** | -0.255 | 0.570* |
| 21. I do something that has made me feel better in the past | Dist | -0.049 | 0.027 | 0.040 | 0.195 | **-0.510** | 0.509 |
| 22. I think about a recent situation, wishing it had gone better | Rum | -0.108 | 0.167 | 0.112 | **0.534** | -0.055 | 0.542 |
| 23. I think “I’m going to go out and have some fun” | Dist | -0.015 | 0.139 | -0.022 | -0.280 | **-0.761** | 0.473 |
| 24. I make a plan to overcome a problem | PS | -0.018 | 0.020 | -0.007 | 0.189 | **-0.528** | 0.478 |
| 25. I stay with other people | PS | -0.035 | -0.104 | 0.169 | -0.185 | **-0.565** | 0.418* |
| 26. I concentrate on my work | Dist | 0.001 | -0.283 | -0.029 | -0.061 | **-0.415** | 0.434 |
| 28. I think about how sad I feel | Rum | 0.050 | -0.086 | 0.211 | **0.732** | 0.126 | 0.638* |
| 29. I think about all my faults and mistakes | Rum | 0.084 | -0.108 | 0.321 | **0.613** | 0.188 | 0.613* |
| 30. I do something I enjoy | Dist | 0.019 | 0.085 | -0.081 | -0.374 | **-0.800** | 0.418* |
| 31. I think about how I don’t feel up to doing anything | Rum | -0.060 | 0.320 | 0.059 | **0.641** | 0.075 | 0.626* |
| 32. I do something that’s fun with a friend | Dist | 0.006 | 0.008 | 0.034 | -0.376 | **-0.813** | 0.451 |
| 33. I think about myself to try to understand why I am depressed | Rum | -0.036 | 0.071 | -0.033 | **0.698** | -0.188 | 0.577* |
| 35. I go somewhere alone to think about my feelings | Rum | 0.113 | -0.063 | -0.063 | **0.771** | 0.076 | 0.601* |
| 36. I think about how angry I am with myself | Rum | 0.140 | -0.035 | 0.221 | **0.616** | 0.102 | 0.629* |
| 37. I listen to sad music | Rum | 0.092 | -0.058 | 0.093 | **0.527** | 0.029 | 0.597* |
| 38. I stay by myself and think about the reasons why I feel sad | Rum | 0.089 | -0.050 | 0.010 | **0.833** | 0.109 | 0.634* |
| 39. I try to understand myself by concentrating on my depressed feelings | Rum | 0.141 | 0.040 | 0.022 | **0.729** | 0.054 | 0.634* |
| **MFQ Items** | **RCMAS Item** |  |  |  |  |  |  |
| 1. I felt miserable or unhappy |  | **0.518** | -0.031 | 0.088 | 0.070 | 0.025 | 0.564* |
| 2. I didn’t enjoy anything |  | **0.293** | 0.207 | -0.113 | -0.038 | 0.037 | 0.499 |
| 3. I was less hungry than usual |  | **0.195** | 0.096 | 0.084 | 0.046 | 0.022 | 0.600* |
| 4. I ate more than usual |  | 0.053 | **0.196** | 0.088 | -0.184 | -0.111 | 0.473 |
| 5. I felt so tired I just sat around and did nothing | RCMAS 21 | 0.127 | **0.408** | -0.085 | 0.082 | 0.058 | 0.557 |
| 6. I was moving and talking more slowly than usual |  | 0.199 | **0.471** | -0.069 | -0.016 | -0.006 | 0.592* |
| 7. I was very restless | RCMAS 33 | 0.124 | **0.268** | 0 | -0.030 | -0.093 | 0.518 |
| 8. I felt I was no good any more |  | **0.651** | 0.088 | 0.067 | 0 | -0.062 | 0.592* |
| 9. I sometimes blamed myself for things that weren’t my fault |  | **0.506** | -0.051 | 0.145 | 0.085 | -0.097 | 0.566 |
| 10. It was hard for me to make up my mind | RCMAS 1 | 0.230 | **0.279** | 0.116 | -0.095 | -0.090 | 0.555 |
| 11. I got cross and grumpy easily |  | **0.354** | 0.155 | 0.164 | -0.031 | -0.068 | 0.652* |
| 12. I felt like talking a lot less than usual |  | 0.306 | **0.319** | -0.051 | 0.123 | 0.051 | 0.629* |
| 13. I was talking more slowly than usual |  | 0.301 | **0.544** | -0.129 | -0.102 | -0.043 | 0.487 |
| 14. I cried a lot |  | **0.608** | -0.186 | 0.134 | 0.067 | -0.124 | 0.571* |
| 15. I thought there was nothing good for me in the future |  | **0.574** | 0.170 | 0.035 | -0.052 | 0.022 | 0.607* |
| 16. I thought that life was not worth living |  | **0.845** | 0.106 | -0.151 | 0.032 | 0.071 | 0.561* |
| 17. I thought about dying |  | **0.793** | 0.028 | -0.152 | 0.074 | 0.042 | 0.571* |
| 18. I thought my family would be better off without me |  | **0.814** | 0.013 | 0.017 | -0.025 | 0.005 | 0.550 |
| 19. I thought about killing myself |  | **0.983** | 0.042 | -0.259 | 0.091 | 0.045 | 0.552* |
| 20. I didn’t want to see my friends |  | **0.305** | 0.110 | 0.003 | 0.163 | 0.049 | 0.579* |
| 21. I found it hard to think properly or concentrate |  | 0.260 | **0.403** | 0.036 | 0.040 | -0.092 | 0.595* |
| 22. I thought bad things would happen to me | RCMAS 37 | **0.427** | 0.101 | 0.207 | -0.107 | -0.099 | 0.531 |
| 23. I hated myself |  | **0.781** | -0.161 | 0.114 | 0.059 | -0.001 | 0.565 |
| 24. I was a bad person |  | **0.511** | 0.096 | 0.147 | -0.109 | 0.057 | 0.560 |
| 25. I thought I looked ugly |  | **0.522** | -0.018 | 0.138 | 0.097 | 0.022 | 0.650* |
| 26. I worried about aches and pains |  | 0.169 | 0.165 | **0.189** | -0.027 | -0.115 | 0.595* |
| 27. I felt lonely | RCMAS 15 | **0.468** | 0.150 | 0.069 | 0.115 | 0.037 | 0.609* |
| 28. I thought nobody really loved me |  | **0.695** | 0.130 | -0.027 | 0.089 | 0.025 | 0.603* |
| 29. I didn’t have any fun at school |  | 0.249 | **0.312** | 0.045 | -0.080 | -0.012 | 0.615* |
| 30. I thought I could never be as good as other kids |  | **0.429** | 0.257 | 0.194 | -0.055 | 0.025 | 0.614* |
| 31. I did everything wrong |  | **0.512** | 0.164 | 0.112 | -0.060 | -0.092 | 0.601* |
| 32. I didn’t sleep as well as usual |  | 0.176 | **0.189** | 0.071 | 0.027 | -0.095 | 0.612* |
| 33. I slept more than usual |  | -0.009 | **0.256** | 0.020 | -0.105 | 0.017 | **0.433** |
| RCMAS Items |  |  |  |  |  |  |  |
| 2. I got nervous when things did not go the right way for me |  | -0.077 | 0.116 | **0.563** | -0.044 | 0.007 | 0.584* |
| 3. Others seemed to do things more easily than I could |  | -0.068 | 0.185 | **0.493** | -0.094 | 0.012 | 0.596* |
| 5. Often I had trouble getting breath |  | -0.135 | 0.122 | **0.431** | -0.068 | 0.015 | 0.555 |
| 6. I worried a lot of the time |  | -0.015 | -0.102 | **0.756** | 0.070 | 0.078 | 0.618* |
| 7. I was afraid of a lot of things |  | -0.044 | -0.019 | **0.738** | 0.001 | 0.044 | 0.595* |
| 9. I got angry easily |  | 0.196 | 0.112 | **0.395** | -0.005 | 0.008 | 0.616* |
| 10. I worried about what my parents would say to me |  | -0.001 | 0.175 | **0.443** | 0.053 | 0.034 | 0.506 |
| 11. I felt that others did not like the way I did things |  | -0.021 | 0.222 | **0.554** | -0.091 | 0.013 | 0.575* |
| 13. It was hard for me to get to sleep at night |  | 0.014 | 0.126 | **0.332** | 0.045 | -0.006 | 0.592* |
| 14. I worried about what other people thought about me |  | 0.055 | -0.048 | **0.611** | 0.140 | 0.043 | 0.600* |
| 17. Often I felt sick |  | 0.013 | 0.046 | **0.480** | -0.031 | -0.005 | 0.593* |
| 18. My feelings got hurt easily |  | 0.200 | -0.233 | **0.621** | 0.157 | -0.025 | 0.635* |
| 19. My hands felt sweaty |  | -0.098 | 0.156 | **0.285** | 0.088 | -0.003 | 0.505 |
| 22. I worried about what was going to happen |  | -0.140 | 0.047 | **0.768** | 0.072 | 0.026 | 0.593* |
| 23. Other children were happier than I |  | 0.108 | 0.050 | **0.471** | 0.045 | 0.123 | 0.593* |
| 25. I have had bad dreams |  | 0.098 | -0.131 | **0.413** | -0.023 | -0.193 | 0.545 |
| 26. My feelings got hurt easily when I was fussed at |  | 0.097 | -0.122 | **0.641** | 0.088 | -0.013 | 0.580* |
| 27. I felt someone will tell me I do things the wrong way |  | -0.016 | 0.203 | **0.555** | -0.020 | -0.006 | 0.577* |
| 29. I wake up scared some of the time |  | 0.035 | -0.022 | **0.563** | -0.084 | -0.184 | 0.529 |
| 30. I worried when I went to bed at night |  | 0.004 | -0.129 | **0.621** | 0.101 | -0.024 | 0.598* |
| 31. It was hard for me to keep my mind on my school work |  | -0.079 | **0.438** | 0.265 | 0.028 | -0.041 | 0.608* |
| 34. I was nervous |  | -0.145 | 0.071 | **0.687** | 0.038 | 0.052 | 0.540 |
| 35. A lot of people were against me |  | 0.059 | 0.240 | **0.443** | 0.006 | 0.101 | 0.579* |
| Mean Factor Loading ^a^ |  | 0.561 | 0.350 | 0.524 | 0.574 | -0.567 |  |
| Factor Determinacy |  | 0.978 | 0.929 | 0.968 | 0.973 | 0.956 |  |

All entries in columns three through seven are estimated factor loadings for questionnaire items on their respective latent factors. MFQ = Mood and Feelings Questionnaire, RDQ = Responses to Depression Questionnaire RCMAS = Revised Children's Manifest Anxiety Scale. 2^nd^ column represents RDQ subscale (Rum = Rumination; Dist = distraction; PS = problem-solving) or whether that MFQ item is also found in the RCMAS. AUC = area under the curve for 3-category (MFQ) or 4-category (RDQ) ordinal items predicting depression diagnosis during follow-up. * 95% confidence interval of AUC does not include 0.5.  ^a^ Mean loading of factor scores for items for which that is the highest-loading factor
