# Supplementary material for: Rumination, anxiety, depressive symptoms and subsequent depression in adolescents at risk for psychopathology: a longitudinal cohort study
Source: BMC Psychiatry. 2013 Oct 8;13:250. doi: 10.1186/1471-244X-13-250 (PMC3851454; doi:10.1186/1471-244X-13-250)
Supplement: Additional file 2: Table S2 — Five factor confirmatory factor analysis of pooled RDQ, MFQ and RCMAS items. [file 1471-244X-13-250-S2.docx]

**Supplementary Table s2 Five factor confirmatory factor analysis of pooled RDQ, MFQ and RCMAS items**

|  | **Factor**  **1**  *Cognitive* | **Factor**  **2**  *Somatic* | **Factor**  **3**  *Anxiety* | **Factor**  **4**  *Rumin-ation* | **Factor**  **5**  *Adaptive* |
| --- | --- | --- | --- | --- | --- |
| RDQ 1 |  |  |  |  | 0.548 |
| RDQ 2 |  |  |  | 0.626 |  |
| RDQ 3 |  |  |  | 0.618 |  |
| RDQ 4 |  | 0.542 |  |  |  |
| RDQ 5 |  | 0.674 |  |  |  |
| RDQ 6 |  |  |  |  | 0.591 |
| RDQ 8 |  |  |  |  | 0.624 |
| RDQ 9 |  |  |  |  | 0.634 |
| RDQ 10 |  |  |  | 0.641 |  |
| RDQ 11 |  |  |  |  | 0.559 |
| RDQ 12 |  |  |  | 0.571 |  |
| RDQ 13 |  |  |  | 0.616 |  |
| RDQ 14 |  |  |  | 0.598 |  |
| RDQ 15 |  |  |  | 0.625 |  |
| RDQ 16 |  |  |  |  | 0.532 |
| RDQ 17 |  |  |  | 0.646 |  |
| RDQ 18 |  |  |  |  | 0.624 |
| RDQ 19 |  |  |  |  | 0.614 |
| RDQ 20 |  |  |  | 0.461 |  |
| RDQ 21 |  |  |  |  | 0.664 |
| RDQ 22 |  |  |  | 0.630 |  |
| RDQ 23 |  |  |  |  | 0.622 |
| RDQ 24 |  |  |  |  | 0.659 |
| RDQ 25 |  |  |  |  | 0.487 |
| RDQ 26 |  |  |  |  | 0.275 |
| RDQ 28 |  |  |  | 0.788 |  |
| RDQ 29 |  |  |  | 0.762 |  |
| RDQ 30 |  |  |  |  | 0.575 |
| RDQ 31 |  |  |  | 0.755 |  |
| RDQ 32 |  |  |  |  | 0.625 |
| RDQ 33 |  |  |  | 0.693 |  |
| RDQ 35 |  |  |  | 0.670 |  |
| RDQ 36 |  |  |  | 0.784 |  |
| RDQ 37 |  |  |  | 0.570 |  |
| RDQ 38 |  |  |  | 0.771 |  |
| RDQ 39 |  |  |  | 0.777 |  |
| MFQ 1 | 0.577 |  |  |  |  |
| MFQ 2 | 0.266 |  |  |  |  |
| MFQ 3 | 0.350 |  |  |  |  |
| MFQ 4 |  | 0.168 |  |  |  |
| MFQ 5 |  | 0.387 |  |  |  |
| MFQ 6 |  | 0.456 |  |  |  |
| MFQ 7 |  | 0.313 |  |  |  |
| MFQ 8 | 0.732 |  |  |  |  |
| MFQ 9 | 0.644 |  |  |  |  |
| MFQ 10 |  | 0.483 |  |  |  |
| MFQ 11 | 0.577 |  |  |  |  |
| MFQ 12 |  | 0.542 |  |  |  |
| MFQ 13 |  | 0.486 |  |  |  |
| MFQ 14 | 0.626 |  |  |  |  |
| MFQ 15 | 0.639 |  |  |  |  |
| MFQ 16 | 0.744 |  |  |  |  |
| MFQ 17 | 0.672 |  |  |  |  |
| MFQ 18 | 0.767 |  |  |  |  |
| MFQ 19 | 0.804 |  |  |  |  |
| MFQ 20 | 0.461 |  |  |  |  |
| MFQ 21 |  | 0.632 |  |  |  |
| MFQ 22 | 0.601 |  |  |  |  |
| MFQ 23 | 0.758 |  |  |  |  |
| MFQ 24 | 0.586 |  |  |  |  |
| MFQ 25 | 0.650 |  |  |  |  |
| MFQ 26 |  |  | 0.434 |  |  |
| MFQ 27 | 0.667 |  |  |  |  |
| MFQ 28 | 0.762 |  |  |  |  |
| MFQ 29 |  | 0.434 |  |  |  |
| MFQ 30 | 0.709 |  |  |  |  |
| MFQ 31 | 0.666 |  |  |  |  |
| MFQ 32 |  | 0.425 |  |  |  |
| MFQ 33 |  | 0.114 |  |  |  |
| RCMAS 2 |  |  | 0.530 |  |  |
| RCMAS 3 |  |  | 0.475 |  |  |
| RCMAS 5 |  |  | 0.342 |  |  |
| RCMAS 6 |  |  | 0.689 |  |  |
| RCMAS 7 |  |  | 0.663 |  |  |
| RCMAS 9 |  |  | 0.601 |  |  |
| RCMAS 10 |  |  | 0.579 |  |  |
| RCMAS 11 |  |  | 0.600 |  |  |
| RCMAS 13 |  |  | 0.455 |  |  |
| RCMAS 14 |  |  | 0.698 |  |  |
| RCMAS 17 |  |  | 0.486 |  |  |
| RCMAS 18 |  |  | 0.722 |  |  |
| RCMAS 19 |  |  | 0.378 |  |  |
| RCMAS 22 |  |  | 0.725 |  |  |
| RCMAS 23 |  |  | 0.576 |  |  |
| RCMAS 25 |  |  | 0.422 |  |  |
| RCMAS 26 |  |  | 0.687 |  |  |
| RCMAS 27 |  |  | 0.652 |  |  |
| RCMAS 29 |  |  | 0.545 |  |  |
| RCMAS 30 |  |  | 0.606 |  |  |
| RCMAS 31 |  | 0.573 |  |  |  |
| RCMAS 34 |  |  | 0.623 |  |  |
| RCMAS 35 |  |  | 0.613 |  |  |
| Mean Factor Loading | *0.632* | *0.445* | *0.570* | *0.633* | *0.576* |

MPlus STDYX standardized estimates are used
